# Evaluation and development of diagnostic tools for rapid detection of *Riemerella anatipestifer* and *Pasteurella multocida* in ducks

**DOI:** 10.5455/javar.2023.j671

**Published:** 2023-06-30

**Authors:** Mohamed M. M. Megahed, Aya M. A. El-Nagar, Azza S. El-Demerdash, Mervat A. Ayoub, Hala M. N. Tolba

**Affiliations:** 1 Avian and Rabbit Medicine Department, Faculty of Veterinary Medicine, Zagazig University, Zagazig, Egypt; 2 Microbiology Department, Agriculture Research Centre (ARC), Animal Health Research Institute (AHRI), Zagazig Branch, Zagazig, Egypt; 3 Pathology Department, Agriculture Research Centre (ARC), Animal Health Research Institute (AHRI), Zagazig Branch, Zagazig, Egypt

**Keywords:** Antibiotic resistance, ducks, multiplex RT-PCR, *Riemerella anatipestifer*, *Pasteurella multocida*

## Abstract

**Objectives:**

Ducks suffer a huge economic loss as a result of infections with *Pasteurella multocida *and* Riemerella anatipestifer*, which cause high morbidity and mortality. Because these pathogens induce similar clinical symptoms when coinfections occur, it is very difficult to differentiate between them based just on clinical signs. Hence, these major pathogens must be quickly and accurately detected.

**Materials and Methods:**

A total of 104 birds ranging from 2 days to 4 weeks old were collected from Egyptian farms, and the outcomes were compared statistically. Conventional cultural identification procedures and a direct multiplex polymerase chain reaction assay were utilized to recognize both pathogens in a single tube reaction simultaneously. Then, the obtained isolates were characterized phenotypically and genotypically.

**Results:**

Clinical signs appear at 2–4 weeks of age with respiratory distress (dyspnea), white fluid feces, and stunting. The scrutinized data demonstrated a significantly higher detection rate by PCR directly compared to classical culture procedures. *Pasteurella multocida* was detected only by PCR. The disc diffusion technique against ten antibiotics showed absolute susceptibilities to amikacin, doxycycline, and florfenicol. High levels of beta-lactam resistance were observed. *Riemerella anatipestifer* isolates were screened for pathogenicity and plasmid-borne* blaTEM* genes. All six isolates harbored five virulence genes: *aspC, RA46, m28, pstS, and Nlp/P60*. Moreover,* blaTEM* was identified into four isolates and deposited to GenBank with accession numbers OP347083, OP347084, OP347085, and OP347086.

**Conclusion:**

These results suggest advanced PCR assays can be applied to the field for rapid and valuable diagnosis of two significant pathogens and focus on the worth of ducks in the propagation of transferable antibiotic resistance genes into the environment.

## Introduction

In the duck industry, bacterial infections are the major contributors to financial losses [1]. Duck mortality is more frequently brought on by bacterial infections than viral ones [2]. Each year, the incidence of duck mortality events and the range of pathogenic bacterial infections that cause that mortality has increased. Many bacterial diseases, particularly *Pasteurella multocida* and *Riemerella anatipestifer*, pose a global danger to duck health. Ducks are susceptible to the contagious and septic disease known as fowl cholera, caused by *P. multocida* [3–5]. It is a significant disease in the duck sector due to the prevalence of *P. multocida* carriers in healthy duck flocks as high as 63% and the potential for 50% mortality [1,6,7].

*Riemerella anatipestifer*-induced duck serositis is the most critical bacterial contagious infection causing acute or chronic disease; the infection period starts from 2 to 7 weeks of age, and its mortality rate reaches 91% [8]. The infections referred to novel duck diseases, anatipestifer syndrome, infectious serositis, and duck septicemia. *Riemerella anatipestifer* is a rod-shaped, Gram-negative, non-spore-forming, non-motile member of the family Flavobacteriaceae of the phylum Bacteroidetes [9]. The chronic form of the disease in birds might result in mucopurulent or caseous salpingitis, which reduces egg production [8].

Molecular techniques such as PCR represent sensitive ways of detecting specific pathogens in field samples and would be quick and affordable to simultaneously detect *P. multocida *and* R. anatipestifer *infections using a multiplex PCR (mPCR) method, which is routinely used to identify several pathogens in clinical samples [1,10,11].

Additionally, evaluating respiratory pathogens for antibiotic susceptibility is a crucial step in determining the best antimicrobial drug to utilize [6,12]. Antibiotic resistance is a significant problem, particularly in human and veterinary medicine. Treatments for diseases may fail as a result of antibiotic resistance. Numerous plasmids harbored these resistance genes and were involved mainly in these phenomena [13]. However, very few reviews have discussed these antibiotic resistance-related variables.

This study was created to investigate the carrier rates *of P. multocida *and* R. anatipestifer* in ducks in the Sharika Provinces using conventional assays or directly from samples using advanced techniques of PCR. In addition, we examined the antimicrobial susceptibilities of these lethal infections in ducklings.

## Materials and Methods

### Ethical approval

The study was conducted with the permission of the Faculty of Veterinary Medicine, Zagazig University, and in line with the committee’s guidelines, with approval number ZU-IACUC/2/F/96/2022.

### Sampling

Between September 2021 and February 2022, a total of 832 internal organs from 104 ducks from eight different districts (backyards and farms) in the Sharkia Governorate—representing a variety of producing sectors, breeds, and ages—were randomly collected. The liver, heart, lung, kidney, brain, air sac, bone marrow, and spleen were the organs from which samples were collected. These samples were then placed in polyethylene bags, labeled, and checked for bacterial prevalence.

### Isolation and identification of R. anatipestifer and P. multocida 

On MacConkey’s agar and sheep blood agar, organs were swabbed right at the entry locations. Blood agar was incubated for 24–72 h at 37°C in an atmosphere that was enhanced with CO_2_ using a candle jar with high humidity, whereas MacConkey’s agar plates were incubated in an aseptic environment [14].

Gram stain was used to colorize smears following standard protocols. The ability of *R. anatipestifer* to liquefy gelatin and its inability to produce indole and ornithine decarboxylase are key differences from *P. multocida* [15]*. *All agars and chemicals were purchased from Oxoid, USA. The pure colonies were then placed in tryptic soy broth with 20% glycerol and kept at −80°C for later analysis.

### Antimicrobial sensitivity test

Using Mueller-Hinton agar plates (Oxoid, UK) enriched with 5% sheep blood, the disc diffusion method was used to determine the antibiotic sensitivity pattern of bacterial isolates to the most potent antibiotics. A 0.5 McFarland standard was used to adjust the final concentration of the bacterial suspension in sterile normal saline before it was swabbed onto the agar plates and incubated for 18–24 h at 37°C in 5% CO_2_ [16]. All isolates were tested for several antibiotics (OXOID) and their concentrations on a per-disc basis as follows: erythromycin (E; 15), ampicillin/sulbactam (SAM; 30), norfloxacin (NOR; 5), neomycin (N; 30), sulfamethoxazole-trimethoprim (SXT; 25), amikacin (AK; 30), and florfenicol (FFC, 30). According to CLSI [17], the results were interpreted. *Riemerella anatipestifer* ATCC 11845 and *P. multocida* ATCC 43137 were used as controls.

### Molecular assay

*Validation of PCR*

Dual-labeled probes were designed using internet-based tools PCR primers and probes were developed for single real-time PCR techniques and verified for specificity and sensitivity previously mentioned [18] in the Biotechnology Laboratory, Animal Health Research Institute, Zagazig Branch, Egypt. The PCR primers and probes were provided by Willowfort (UK) and listed in Table 1.

### DNA and plasmid extraction

DNA was extracted directly from samples and from bacterial cultures following the manufacturer‘s instructions for QIAamp DNA Mini kits (Qiagen, Germany, GmbH, Catalogue No. 51304). Plasmid DNAs were extracted from bacterial isolates using Plasmid DNA Miniprep Kits (Thermo Fisher Scientific, Waltham, MA) following the manufacturer’s instructions and screened for the presence of the beta-lactamase genes.

### Multiplex Tag man real-time PCR amplification

The final volume for the PCR reaction was 20 μl, which included 10 μl of 2× Sensifast probe No-ROX buffer (Bioline, UK), 3.75 μl of PCR grade water, 0.25 μl of each primer (50 pmol conc.), 0.125 μl of each probe (30 pmol conc.), and 5 μl of DNA template. Each run has included negative (PCR master mix without DNA template) and positive controls. The cycling conditions were: 95°C for 10 min, followed by 35 cycles of initial denaturation at 95°C for 45 sec; primer annealing (TA) for 45 sec; primer extension at 72°C for 10 min; and a final holding temperature of 4°C (Table 1). The melting curve analysis and fluorescence intensity were assessed; a threshold cycle (Ct) under 35 and a specific melting temperature (Tm) indicated a positive result.

**Table 1.** Oligonucleotide primers and probes used in this study for PCR assays.

Target gene	Sequence (5’-3’)	TA (°C)	Reference
*P. multocida* 16S rRNA	ATCCGCTATTTACCCAGTGG GCTGTAAACGAACTCGCCAC P: (VIC)TTGATGCCTTCTTTGCGGGTTTCG	55	[38]
*R. anatipestifer* 16S rRNA	TATTTTATTTTTGTGTCTATGAACT TCTTGGCTGAGTTTTAATCT P: (FAM) CGGTTACCATCATAGAAGCGTCAA	55	[39]
*aspC*	CGTCGTCTATAAGAGCGGCTAA GGGGAACCCGATTTTGATGT	60	[40]
*RA46*	AGCATCATTAGTGCGTATCTCAA CCCTTCCCTCTTTATCCATTT 187	60	[40]
*m28*	TTTCCCAAGAACGCCACTCA CCCTAAAATGCAACAAGCTCAC	60	[40]
*pstS*	AGTGCTACCAGTGATGGATGA ATCCATTCCCAACCCCGAAA	60	[37]
*hydrolase Nlp/P60*	GCGTTGTAAGCGGCTTTACT ACTCACTGCCGCTCATAAGA	60	[37]
*blaTEM*	ATCAGCAATAAACCAGC CCCCGAAGAACGTTTTC	54	[41]

### Conventional PCR amplification for virulence and blaTEM genes

The final volume for the PCR cycling operation was 25 μl which included 12.5 μl of DreamTaq Green PCR Master Mix (2×) from Thermo Scientific, 1 μl of each primer at a concentration of 20 pmol, 5.5 μl of water, and 5 μl of DNA template. The carried-out reaction was in an Applied Biosystem 2720 thermal cycler under the following cycling conditions: 94°C for 5 min, followed by 35 cycles of initial denaturation at 94°C for 30 sec, primer annealing (TA) as stated in Table 1 for 40 sec, and primer extension at 72°C for 7 min.

### Sequence analysis

An automated DNA sequencer (Applied Biosystems 3130, ABI, 3130, USA) was employed to sequence forward and reverse purified PCR products. Utilizing a ready-to-use Bigdye Terminator V3.1 cycle sequencing kit (Cat. No. 4336817, Perkin-Elmer/Applied Biosystems, Foster City, CA), A BLAST^®^ analysis (Basic Local Alignment Search Tool) [19] was initially carried out to determine sequence identity for GenBank accessions. The sequence reactions were carried out in accordance with the manufacturer’s instructions. A phylogenetic tree has formed as the outcome of our sequence analysis of the plasmid strings using Molecular Evolutionary Genetics Analysis (Mega X software).

### Statistical analysis

The data were edited in Microsoft Excel (Microsoft Corporation, Redmond, WA). A binary logistic regression [20] was run to examine the potential risk factors, including breed, age, and season, involved in the risk of *Riemerella* and/or *Pasteurella* detection by traditional methods and RT-PCR. Significant differences between explanatory variables were tested using Fisher’s exact test. Statistical significance was set at a* p*-value less than 0.05. Figures were fitted by the GraphPad Prism software 5.0 (Graph Pad, USA). Diagnostic tests, including sensitivity, specificity, positive and negative predictive values, and positive and negative likelihood ratios, were calculated according to the following functions:

Sensitivity = [a/ (a + c)]× 100

Specificity = [d/ (b + d)] × 100

Positive predictive value = [a/ (a + b)] × 100

Negative predictive value = [d/(c + d)] × 100.

Positive likelihood ratio = Sensitivity/ (1− Specificity).

Negative likelihood ratio = (1 − Sensitivity)/ Specificity.

where a is the true positive case, b is the false positive, c is the false negative, and d is the true negative.

## Results

### Clinical and post-mortem findings

The affected ducklings showed depression, anorexia, mucous discharge from the mouth and nostrils, diarrhea, and increases in respiratory rate. Some ducklings had a history of nervous manifestations (lameness, twisting head and neck, leg paddling, and ataxia). Necropsy findings showed parenchymatous congestion and pneumonia with polyserositis (fibrinous air sacculitis, perihepatitis, and pericarditis). The livers were swollen, accompanied by multiple small necrotic foci.

### Risk factors for Riemerella and/or Pasteurella detection

**Figure 1.**
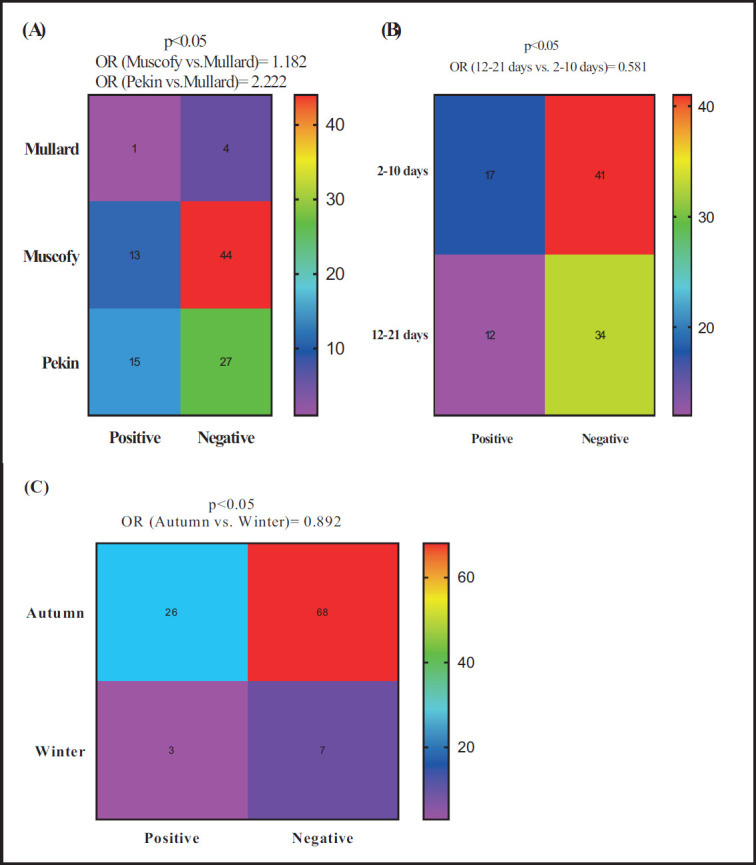
Potential risk factors comprehensive breed (A), age (B), and season (C) associated with the probability of detecting *Riemerella* by the traditional method

**Figure 2.**
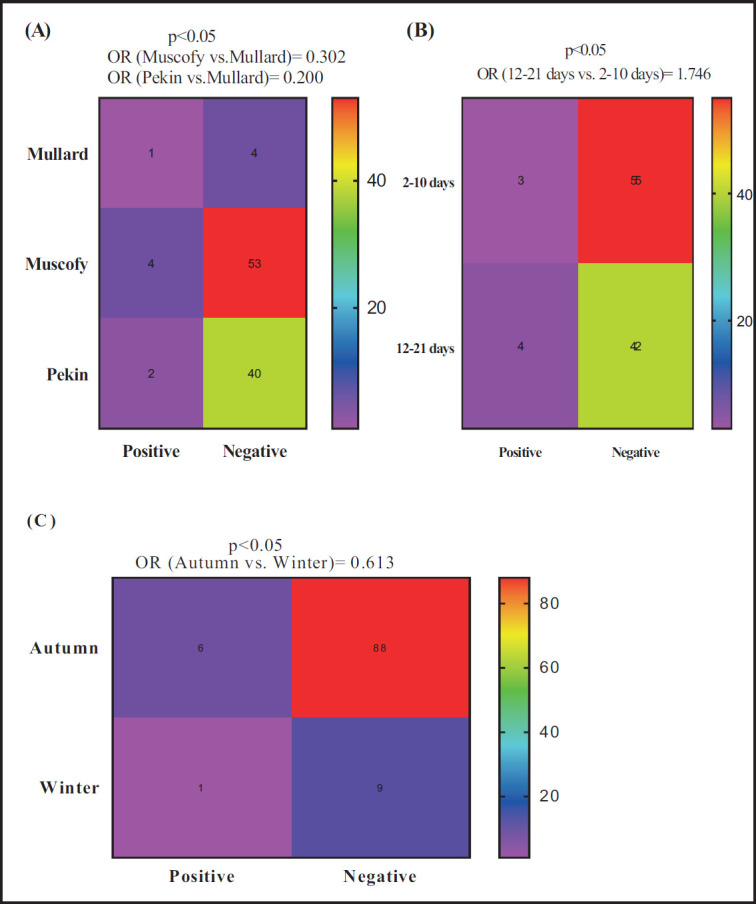
Potential risk factors comprehensive breed (A), age (B), and season (C) associated with the probability of detecting *Pasteurella* by RT-PCR

**Figure 3.**
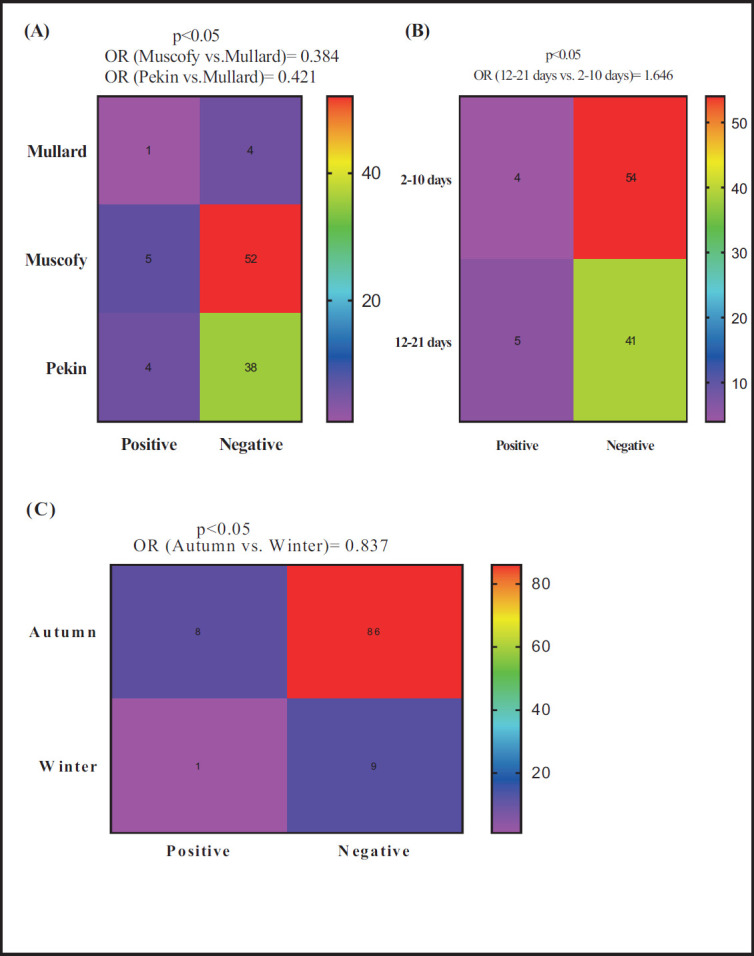
Potential risk factors comprehensive breed (A), age (B), and season (C)associated with the probability of detecting *Riemerella* by RT-PCR

**Figure 4.**
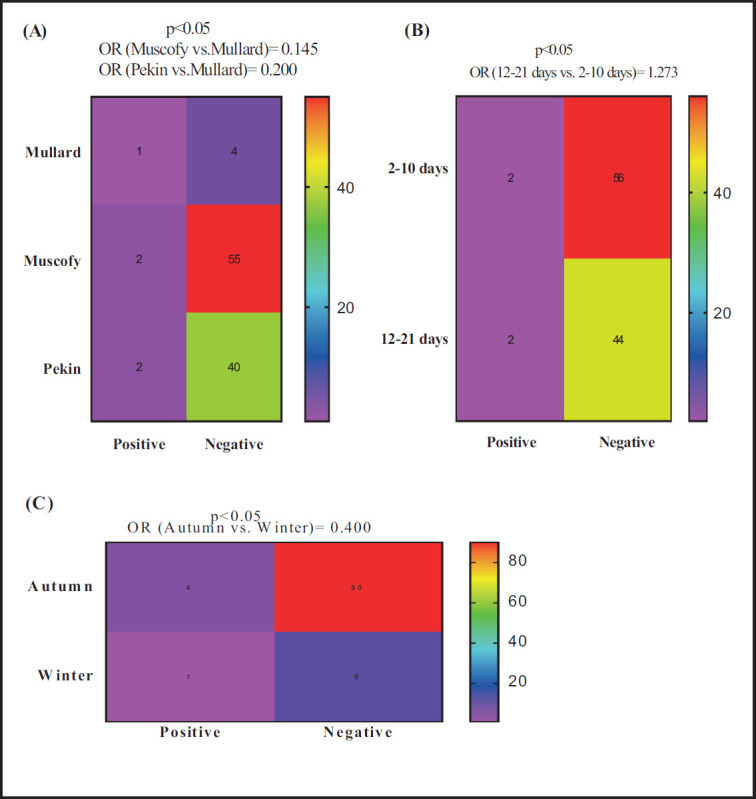
Potential risk factors comprehensive breed (A), age (B), and season (C) associated with the probability of detecting *Pasteurella* plus *Riemerella* by RT-PCR

**Figure 5.**
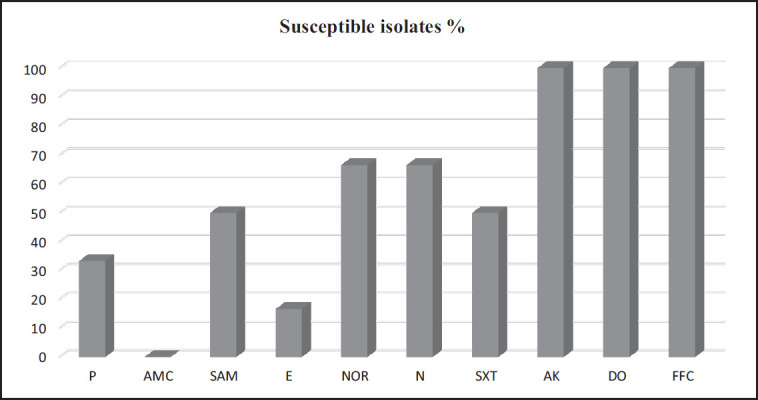
Frequency of antimicrobial susceptibility of *Riemerella anatipestifer *isolates from ducklings. P: Pencillin G, AMC: Amoxicillin-clavulanic acid, SAM: Ampicillin/sulbactam, E: Erythromycin, NOR: Norfloxacin, N: Neomycin, SXT: Sulfamethoxazole-trimethoprim, AK: Amikacin, DO: Doxycycline, FFC: Florfenicol.

The potential risk factors associated with the probability of *Riemerella *and/or *Pasteurella *detection in ducks through the traditional method and RT-PCR are illustrated in Figures 2–5. Herein, by using the conventional assays, the odds of *Riemerella* detection were higher than 18.2% in Muscovy (OR = 1.182) and 2.222 times in Pekin ducks (OR = 2.222) compared to Mullard (Fig. 1a). Age was another risk factor associated with *Riemerella*; the detection probability of *Riemerella* decreased by 41.9% (OR = 0.581) for ducks in the age category 12–21 days compared to those in the age category 2–10 days (Fig. 1b). Compared with the winter season, the probability of *Riemerella* detection decreased by 10.8% (OR = 0.892) during the autumn season (Fig. 1c). However, *P. multocida* was not detected by conventional assays.

Interestingly, by using RT-PCR, the odds of *Pasteurella* detection were lower in both Muscovy (OR = 0.302; 69.8%) and Pekin (OR = 0.200; 80.0%) ducks compared to Mullard (Fig. 2a). Meanwhile, the probability of giving positive cases increased by 74.6% (OR = 1.746) for ducks in the age category 12–21 days compared to those in the age category 2–10 days (Fig. 2b). Similarly, during the autumn season, the odds of *Pasteurella* detection decreased by 38.7 (OR = 0.613) compared to the winter season (Fig. 2c).

For *Riemerella* detection by RT-PCR method, Muscovy and Pekin breeds were 61.8 (OR = 0.382) and 57.9% (OR = 0.421) less likely to show positive cases of *Riemerella* than the Mullard breed (Fig. 3a). The age category of 12–21 days was associated with higher odds (OR = 1.642; 64.2%) of *Riemerella* detection compared to the denomination of 2–10 days (Fig. 3b), while the autumn season was associated with a lower likelihood (OR = 0.837; 16.3%) compared to the winter season (Fig. 3c).

Concerning *Riemerella* plus *Pasteurella* detection via RT-PCR, Muscovy and Pekin breeds had 85.5% (OR = 0.145) and 80% (OR = 0.200), respectively, diminishing odds of positive case detection compared to Mullard (Fig. 4a). Further, ducks in the age category of 12–21 days had 27.3% (OR = 1.273) higher odds of positive case detection than those in the age category of 2–10 days (Fig. 4b). Meanwhile, the probability of detection decreased by 60% (OR = 0.400) during the autumn season compared to the winter (Fig. 4c).

### Diagnostic tests

The illustrated results of diagnostic tests (traditional method and RT-PCR) for *RA* detection are in Tables 2–4. There is a risk that tests with high specificity will capture some individuals who do not have positive cases. Using RT-PCR as the gold standard, the sensitivity is 25% and the specificity is 72%, meaning that the traditional method will correctly identify 25% of the individuals who have positive cases. But it fails to reach 75%. This method will correctly identify 72% of individuals who do not have a positive incidence, but it will also identify 28% of individuals as having a positive case when they do not. Positive and negative predictive values were 3.44 and 96.00%, respectively, which means that the event that the method makes a prediction giving a positive result under the gold standard was 3.44. The event that made no predictions under the gold standard was 96%. Positive and negative likelihood ratios were 89.28 and 1.04%, respectively.

### Antibiogram profile of R. anatipestifer isolates

The findings revealed the antibiotic sensitivity pattern of *R. anatipestifer *isolates and the resistance breakpoints of the antibiotics used in this study. The six *R. anatipestifer* isolates represented a clear sensitivity profile to three antibiotics comprising amikacin, doxycycline, and florfenicol. All the *R. anatipestifer* isolates represented an expanding resistance pattern to amoxicillin-clavulanic acid. More than 50% of isolates exhibited resistance to ampicillin/sulbactam, sulfamethoxazole-trimethoprim, penicillin, and erythromycin. Moreover, 100% of the tested isolates displayed multidrug resistance (MDR) (Fig. 5).

### Genotypic characterization of R. anatipestifer isolates

**Figure 6.**
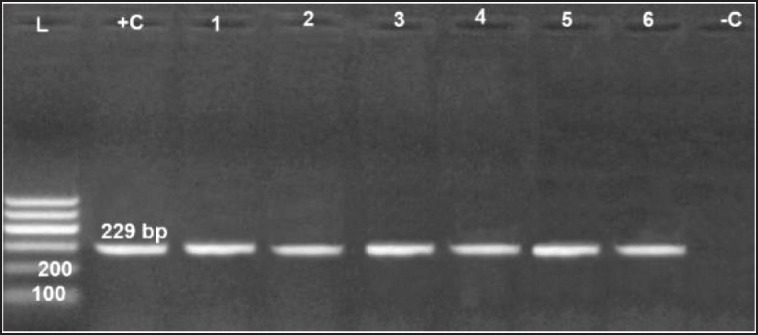
Agarose gel electrophoresis of PCR for amplification products of *aspC* gene among six *Riemerella anatipestifer* isolates; Lane +C: Control positive, Lane L: 100-bp ladder (marker); Lane -C: Control negative*.*

**Figure 7.**
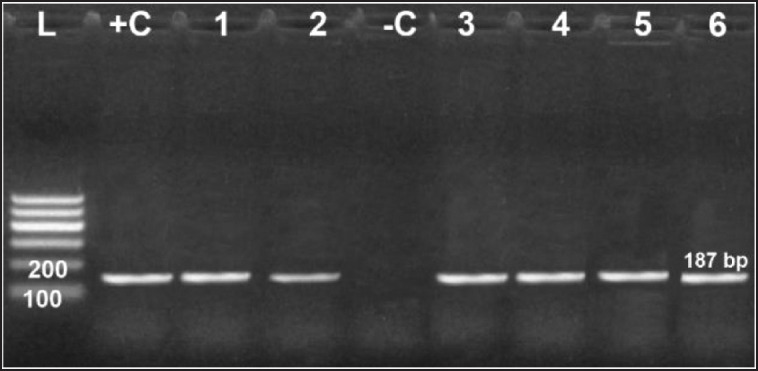
Agarose gel electrophoresis of PCR for amplification products of *RA46 *gene among six *R. anatipestifer* isolates; Lane +C: Control positive, Lane L: 100-bp ladder (marker); Lane -C: Control negative*.*

**Figure 8.**
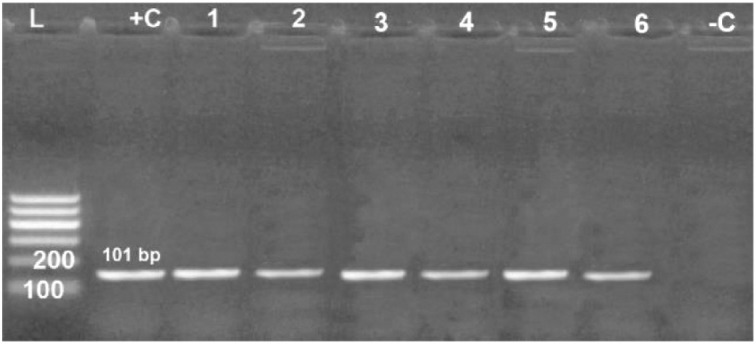
Agarose gel electrophoresis of PCR for amplification products of *m28 *gene among six *R. anatipestifer* isolates; Lane + C: Control positive, Lane L: 100-bp ladder (marker); Lane -C: Control negative*.*

**Figure 9.**
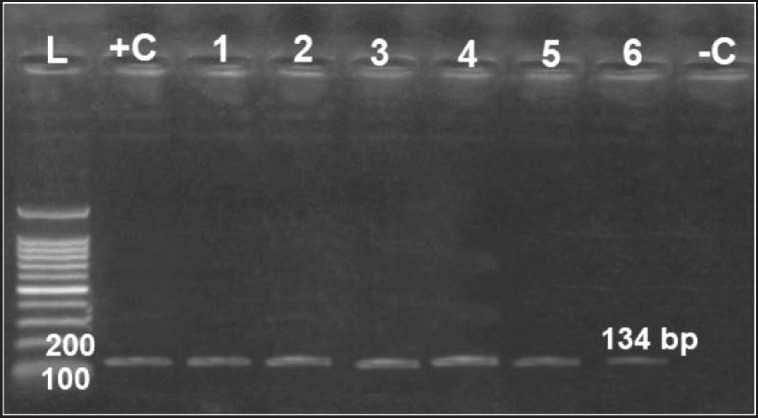
Agarose gel electrophoresis of PCR for amplification products of *pstS* gene among six *R. anatipestifer* isolates; Lane +C: Control positive, Lane L: 100-bp ladder (marker); Lane -C: Control negative*.*

**Figure 10.**
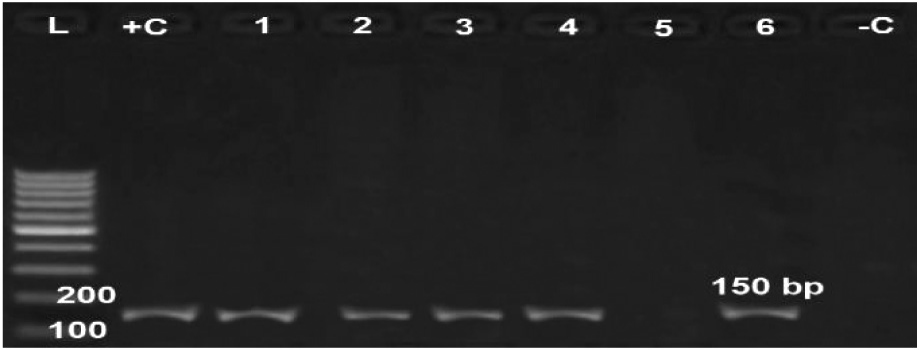
Agarose gel electrophoresis of PCR for amplification products of *hydrolase Nlp/P60 *gene among six *R. anatipestifer* isolates; Lane + C: Control positive, Lane L: 100-bp ladder (marker); Lane -C: Control negative*.*

All examined virulence genes were expressed strongly in six obtained isolates at their specific base pair, except the hydrolase* Nlp/P60 *clone, which was not detected in only one isolate (Fig. 6–10).

*Detection of the β-lactamase gene *(*blaTEM*) *in* *R. anatipestifer isolates and sequencing data*

**Figure 11.**
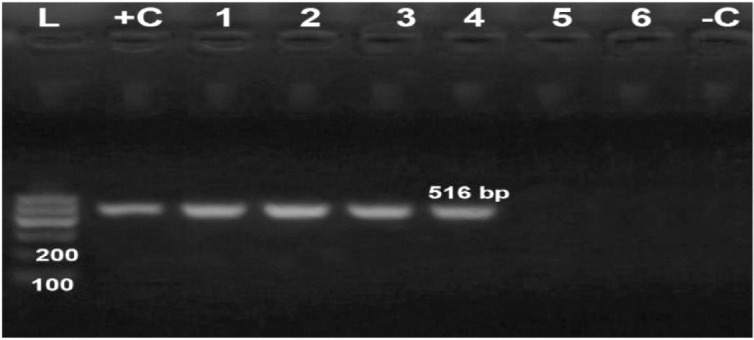
Agarose gel electrophoresis of PCR for amplification products of *bla TEM* gene among six *R. anatipestifer* isolates; Lane + C: Control positive, Lane L: 100-bp ladder (marker), Lanes 1-4: Positive samples for *bla TEM* gene; Lane -C: Control negative*.*

Conventional PCR amplification revealed that 4 out of 6 (66.6%) of the obtained multidrug-resistant *R. anatipestifer* isolates harbored the *blaTEM* gene, giving an amplicon size of 516 bp, as shown in Figure 11.

We sequenced four gene fragments of *blaTEM *from four *R. anatipestifer* isolates. The detailed amino acid substitutions in the amplified fragments were deposited in GenBank under accession numbers OP347083, OP347084, OP347085, and OP347086.

**Table 2.** Molecular prevalence % of *P. multocida *and* R. anatipestifer* from different districts concerning different ages and breed.

Localities	Examined farms	Examined cases (*n* = 104)	Breed	Age/ Week	Morbidity	Mortality	*P. multocida* Positive* (%)	*R. anatipestifer* Positive* (%)
Belbeis	1	15	Pekin	3	50	20	1 (6.6)	4 (26.6)
Bardein	2	15	Muscovy	3	30	20		3 (20)
		14		2	25	10	1 (7.1)	1 (7.1)
Derb Negm	1	14	Muscovy	2	25	20		2 (14.2)
Minea El-Kamgh	2	21	Pekin	2	50	10		
		15	Muscovy	3	33	15		
Abu Kabir	1	5	Mallard	4	41	3	3 (60)	3 (60)
Fakous	1	5	Pekin	2	30	50		

The result was highly significant difference at *p *< 0.05

**Table 3.** Number of subjects in positive cases of *R. anatipestifer* was detected using RT-PCR (the gold standard) versus the results of the traditional method.

Total	Results of RT-PCR as a gold standard		Result of test under evaluation
	Negative	Positive	
29	b (False positive = 28)	a (True positive = 1)	Positive
75	d (True negative = 72)	c (False negative = 3)	Negative
104	100	4	Total

**Table 4.** Analysis of diagnostic tests (Traditional method and RT-PCR as a gold standard test) for *RA* detection

Parameters	Estimates (%)
Sensitivity	25.00
Specificity	72.00
PV+	3.44
PV –	96.00
LR+	89.28
LR –	1.04

PV refers to Prediction Value; LR refers to Likelihood ratio

**Figure 12.**
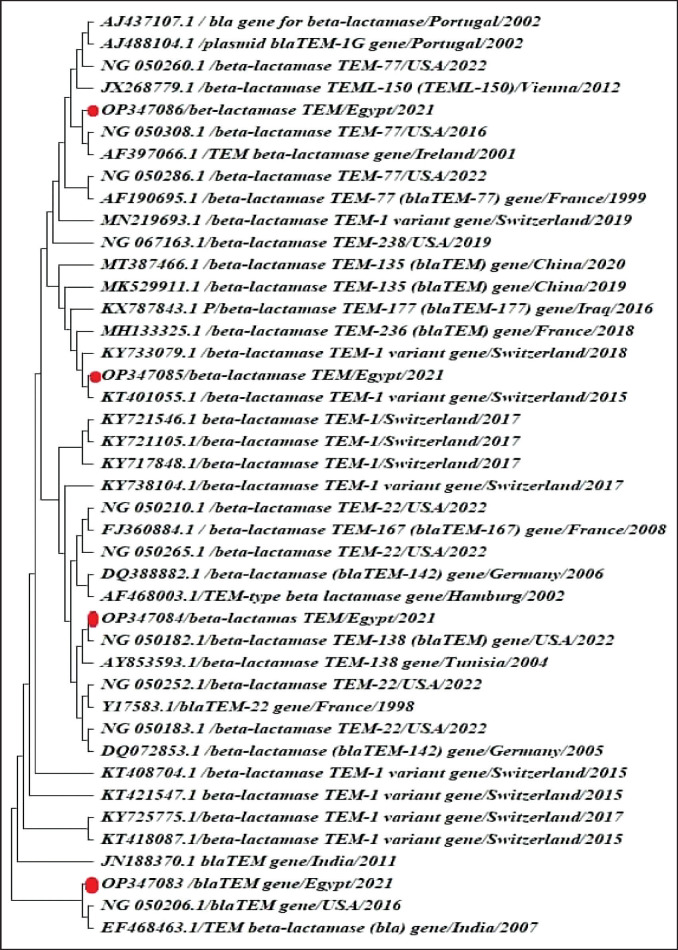
Phylogenetic tree of the different *blaTEM *clades. The 4 MDR *R. anatipestifer* isolates of the sector used in this analysis were pointed with a red circle

Phylogenetic analysis results of plasmid fragments of *the blaTEM* gene revealed that our isolates are closely related to Portuguese, Indian, and Egyptian isolates, forming clusters with these isolates. Our data are the first report on the prevalence of these genes in *R. anatipestifer *(Fig. 12).

## Discussion

After post-mortem inspection, there were fibrinous exudates in the pericardial cavity and all over the surface of the liver. Airsacculitis with well-organized yellow casts was also observed. All these distinguishing characteristics have been detected in several avian species previously [21]. Initially, post-mortem lesions from the pericardium, air sacs, and liver were subjected to standard methods of microbial isolation in suitable agar media. A bacteriological analysis of 104 birds in a suitable microaerophilic environment revealed the incidence of six positive isolates. *Riemerella anatipestifer* is the term given to bacterial strains with typical non-hemolytic colony, Gram-negative and bipolar staining reactions, and non-motile short rods. Surya et al*.* [22] also identified similar cultural, morphological, and staining features.

Infection with *R. anatipestifer* and *pasteurellosis* in ducks are frequently confused. Hence, to prevent severe mortality, a precise and early diagnosis of this infection is crucial. Since Riemerella species lack distinctive morphological features that indicate difficulties, identification based on cultural and biochemical traits is time-consuming and labor-intensive. PCR is a fast, accurate, and specific method to identify microbial infections [23]. In this study, we used PCR to detect *Riemerella *organisms directly on the obtained samples and confirm the identity of the isolates. This helps in implementing early treatment and control. Interestingly, a higher prevalence of *RA* was detected directly by molecular techniques (*n =* 13). *Riemerella anatipestifer*’s isolation rate is extremely low due to the lack of selective media, specific growth demands, and a variety of phenotypic traits [21].

Multiplex PCR is a potent method in clinical microbiology that has been extensively used to pinpoint genes and pathogens of interest [24]. Because comparable clinical symptoms and coinfections occur in duck flocks, a novel mPCR approach was designed to simultaneously identify and discriminate between *P. multocida* and *R. anatipestifer*, the two most significant bacterial pathogens of ducks.

A co-infection of duck plague and *R. anatipestifer* was detected in five ducks with *P. multocida.* Secondary infections of *P. multocida* in naturally occurring epidemics of duck plague in ducklings may be due to an immunosuppressive state brought on by the disease, according to some experts [1], giving an overall prevalence of 12.5% of *R. anatipestifer *(*n =* 13) and 4.8% of* P. multocida *(*n =* 5). This isolation rate was lower than the level reported by El-Hamid et al. [25] and Shalaby et al. [6]. While higher prevalence rates reaching 50 % were detected in China and India [26]. This difference may be attributed to the variations in ducks’ age and breeds, sample number, isolation protocol, stress, geographical location, and resistance power of ducklings due to management, vaccine, and nutrition.

According to the current research, there were substantial differences in the prevalence of *P. multocida *and* R. anatipestifer *among duck breeds, ages, and seasons. In the study area, Muscovy breeds of ducks had a higher incidence than Pekin and Mallard variants. The variations in genetic resistance to the infection may be responsible for the differences in prevalence between the breeds [2,5].

Regarding seasons and ages, younger birds were more susceptible to infection than older ones, especially in the winter. The immune responses were predominantly responsible for these findings [6,25].

Due to the extensive genetic diversity of the *R. anatipestifer* strains and the low cross-protection between them, the primary treatment for their infection is antimicrobial therapy [27]. As a result, selecting the appropriate antibiotic for a specific situation with *R. anatipestifer* requires conducting in vitro drug sensitivity tests. In numerous studies performed over the period, a variety of antimicrobial treatments have been employed to control the infection of *R. anatipestifer* and reduce the large economic losses at the field level [28].

In our study, six *R. anatipestifer* isolates were tested versus ten antibiotics, widely used agents in the poultry industry. Amikacin, florfenicol, and doxycycline were the drugs of choice for the tested isolates. These results followed those of Priya et al. [29] and Surya et al. [22]. Unfortunately, the indiscriminate use of antimicrobials in feed, both to promote growth and as a preventive strategy, led to significant resistance to almost all practical antibiotics.

There have been reports of the clonal proliferation of *R. anatipestifer* strains in duck farms in Egypt [30, 31]. All *R. anatipestifer* isolates were multidrug resistant in our investigation. Drug resistance rates also tend to rise over time. In detail, multi-drug resistance proportions were 0%, 6.6%, 38.7%, 20.8%, 18%, 30.5%, 55.3%, and 77% for isolates identified in 2000, 2004–2006, 2009–2010, 2011, 2012, 2013, 2014, and 2015–2020, respectively. These findings concur with those of Nhung et al. [32], who recorded a gradual increase in the multi-drug resistance phenomenon over time.

The evolution of resistance genes that encode the drug targets in *R. anatipestifer *has long been linked to the emergence of drug resistance. Based on our clinical investigations, *R. anatipestifer *isolates represented a considerable resistance pattern to beta-lactam antibiotics. Thus, we focused our study on *the blaTEM* genes, which were detected among our *R. anatipestifer* isolates with a percentage of 66.6% and deposited to GenBank, proven as a powerful phylogenetic marker [33]. The genetic similarity observed with the several isolates may be due to the spread of the bacteria through migratory waterfowl and the sharing of the international boundary between the countries [34]. Our isolates harbored this gene on a plasmid vector characterized by its rapid diffusion. This explains the phylogenetic analysis as these genes may have been inserted into the *R. anatipestifer* plasmid from other bacterial infections. These results reflect a great hazard to public health [35].

Of interest, all isolated *R. anatipestifer* harbored understudy genes that have different metabolic pathways to acquire nutrients during growth in the ducklings and maintain the efficiency of energy manufacture to retain maximal growth rates, division, autolysis, and invasion, indicating their profound pathogenicity [36,37].

## Conclusion

The results of the current study demonstrated that PCR assays make it easier to rapidly and precisely identify *R. anatipestifer* infection in ducks. This innovative assay can easily differentiate between *R. anatipestifer* and *P. multocida* and substitute for the traditional protocols that are difficult and time-consuming. A further advantage of PCR assays is their direct detection of *Riemerella* organisms in clinical material. Moreover, early and confirmatory identification of *R. anatipestifer* infection in Egyptian ducks helps in viable vaccine synthesis, which may provide economic relief to commercial duck farmers in Egypt.

## List of abbreviations

*P. multocida*, *Pasteurella multocida*; *R. anatipestifer*, *Riemerella anatipestifer*; real-time polymerase chain reaction; RT-PCR
